# Nerve conduction in relation to vibration exposure - a non-positive cohort study

**DOI:** 10.1186/1745-6673-5-21

**Published:** 2010-07-19

**Authors:** Helena Sandén, Andreas Jonsson, B Gunnar Wallin, Lage Burström, Ronnie Lundström, Tohr Nilsson, Mats Hagberg

**Affiliations:** 1 Occupational and Environmental Medicine, Sahlgrenska School of Public Health and Community Medicine, University of Gothenburg, Sweden; 2 Institute of Neuroscience and Physiology, Sahlgrenska Academy, University of Gothenburg, Sweden; 3 Occupational and Environmental Medicine, Department of Public Health & Clinical Medicine, Umeå University, Sweden; 4 Sundsvall Hospital, Department of Occupational and Environmental Medicine, Sundsvall Hospital, Sweden

## Abstract

**Background:**

Peripheral neuropathy is one of the principal clinical disorders in workers with hand-arm vibration syndrome. Electrophysiological studies aimed at defining the nature of the injury have provided conflicting results. One reason for this lack of consistency might be the sparsity of published longitudinal etiological studies with both good assessment of exposure and a well-defined measure of disease. Against this background we measured conduction velocities in the hand after having assessed vibration exposure over 21 years in a cohort of manual workers.

**Methods:**

The study group consisted of 155 male office and manual workers at an engineering plant that manufactured pulp and paper machinery. The study has a longitudinal design regarding exposure assessment and a cross-sectional design regarding the outcome of nerve conduction. Hand-arm vibration dose was calculated as the product of self-reported occupational exposure, collected by questionnaire and interviews, and the measured or estimated hand-arm vibration exposure in 1987, 1992, 1997, 2002, and 2008. Distal motor latencies in median and ulnar nerves and sensory nerve conduction over the carpal tunnel and the finger-palm segments in the median nerve were measured in 2008. Before the nerve conduction measurement, the subjects were systemically warmed by a bicycle ergometer test.

**Results:**

There were no differences in distal latencies between subjects exposed to hand-arm vibration and unexposed subjects, neither in the sensory conduction latencies of the median nerve, nor in the motor conduction latencies of the median and ulnar nerves. Seven subjects (9%) in the exposed group and three subjects (12%) in the unexposed group had both pathological sensory nerve conduction at the wrist and symptoms suggestive of carpal tunnel syndrome.

**Conclusion:**

Nerve conduction measurements of peripheral hand nerves revealed no exposure-response association between hand-arm vibration exposure and distal neuropathy of the large myelinated fibers in a cohort of male office and manual workers.

## Background

Peripheral neuropathy is one of the principal clinical disorders in workers with hand-arm vibration syndrome (HAVS). In vibration-associated neuropathy, conceivable target structures could be peripheral sensory receptors, large or thin myelinated nerve fibers, and the small-caliber, non-myelinated C fibers. Electrophysiological studies aimed at defining the nature of the vibration injury have provided conflicting results [1]. Fractionated nerve conduction velocity of the median nerve across the carpal tunnel on vibration-exposed subjects with hand symptoms has revealed a bimodal velocity distribution, suggesting effect both at the carpal tunnel and at a more distal level, such as palm or finger [2]. Abnormalities that appear to be independent of clinical entrapment neuropathy have been recognized, and a distal pattern of delayed sensory nerve conduction localized at the digits has been described [3,4]. Pathological studies by cutaneous biopsy have demonstrated demyelinating neuropathy in the digital nerves of individuals with HAVS [5]. On the other hand, Lander et al. found that median and ulnar neuropathies proximal to the hand are more common than digital neuropathies in hand-arm vibration exposed workers with neurological symptoms [6]. However, in a 17-year prospective study of industrial workers, Nathan et al.[7] reported that workplace factors, including managing vibratory tools, appeared to bear an uncertain relationship to carpal tunnel syndrome and Cherniack et al. [8] reported that the significant differences in digital sensory conduction velocities between vibration-exposed and non-exposed workers were eliminated after systemic warming.

One reason for this lack of consistency might be the sparsity of published longitudinal etiological studies which include both a good assessment of exposure and a well-defined measure of disease. In occupational studies that require specification of previous exposure there is always a risk of recall bias. To get a better understanding of exposure-response relationships, it would be desirable to have a longitudinal study design to obtain a more accurate exposure assessment.

The aim of the present study was to assess the possible reductions in median and ulnar nerve conduction velocities in hand-arm vibration exposed workers compared to unexposed workers. To this end, we measured the motor and sensory conduction velocities after having assessed vibration exposure over 21 years in a cohort of manual workers.

## Materials and methods

The study design was cross-sectional regarding the outcome of nerve conduction but longitudinal regarding exposure assessment. The cohort studied was recruited in 1987 and 1992 and has since been followed and assessed for vibration exposure. Ethical approval was obtained by the Regional Ethics Committee in Umeå (Dnr 07-161M).

### Subjects

The cohort consisted of male office workers and male manual workers, all full-time employees at an engineering plant that manufactured pulp and paper machinery. The subjects were recruited from the plant's payroll rosters in two stages: 151 subjects from the roster of January 1, 1987 and 90 subjects from that of January 31, 1992. An upper age limit of 55 years was set for inclusion. From the 1987 roster, 61 of 500 male office workers, including salesmen, managers, engineers, secretaries, and economic clerks, were randomly invited into the study. At the baseline examination in February 1987, 93 of 112 manual workers, including welders, grinders, turners, and steel platers, were available for invitation. Three manual workers declined to enter the study. A total of 151 subjects, 61 office workers and 90 manual workers, were examined and entered the cohort in 1987. In 1992, an additional 33 randomly invited office workers and 57 more manual workers who had been hired after 1987 were examined and added to the cohort (none of the invited subjects declined). Thus, in 1992 the cohort (baseline) consisted of 241 subjects.

Follow-ups were conducted in 1997, 2002, and 2008, i.e. 10, 15, and 21 years after recruitment of the original cohort. At the 10-year follow-up the study group consisted of 220 subjects (9% loss from baseline); at 15 years there were 195 subjects (19% loss from baseline), and at the 21-year follow-up 197 subjects (18% loss from baseline) remained in the cohort (Table 1). The subjects that were lost to follow-up and the returners have been analyzed for age and exposure. They did not differ from those not lost to follow-up. The exposure assessment at baseline revealed that some of the office workers had formerly been exposed to hand-arm exposure and some manual workers were not currently exposed to hand-arm vibration. To simplify, we used the terms exposed, currently exposed and unexposed subjects in the presentation of the study population (Table 1).

**Table 1:** Study population at baseline and follow ups, 1987-2008.

		**1987**	**1987-1992^a^**	**1997**	**2002**	**2008**
	
Total		151	241	220	195	197
	Exposed^d^	112(83)	181(108)	165(90)	141(57)	146(52)
	Unexposed	39	60	55	54	51
Returners from baseline (1987-1992)^c^	Exposed^d^				8(1)	26(13)
	Unexposed				3	2
Lost to Follow up	Exposed^e^		9^b^(7)	16(12)	32(22)	21(4)
	Unexposed		3^b^	5	4	5

^a ^Baseline 1987-1992. Baseline consists of subjects entering the study in 1987 and 1992.

^b ^Lost to follow up between 1987 and 1992. The subjects are included in baseline (n = 241).

^c ^Subjects who were included at baseline, lost to follow up, but returned later to the study group in 2002 and/or 2008.

^d ^Subjects that currently are or previously have been exposed, the currently exposed in brackets.

^e ^Subjects that currently are or previously have been exposed, the currently exposed (based on the latest study) in brackets.

In 2008, all 197 subjects were invited to participate in nerve conduction measurements and 163 subjects were finally examined (83%). The most common reasons for not attending the nerve conduction measurements were that the subjects had retired or moved away from the area. Six subjects were excluded due to diabetes and two subjects due to polyneuropathy. Thus, the nerve conduction study group consisted of 155 subjects.

Five subjects reported a history of carpal tunnel release in the right hand and one subject in the left hand. These hands were also excluded. In some subjects reliable measurements were not obtained due to electromagnetic interference, and some measurements were discontinued because of discomfort. Therefore, the final material of motor conduction measurements consisted of 150 right hands and 148 left hands for the median nerve and 152 right hands and 148 left hands for the ulnar nerve. Median sensory conduction measurements were made in 105 right and 99 left hands.

### Medical examination and questionnaire

Each subject was interviewed regarding symptoms and examined by a physician (T.N). A standard procedure was followed for physical examination of the neuromuscular and skeletal systems of the upper extremities in order to check for and identify other diseases, primarily polyneuropathy. The subjects provided supplementary basic data through a questionnaire. The questions covered age, work and years at work, exposure, chronic disease, symptoms, and use of nicotine and/or alcohol (Table 2).

**Table 2:** Study population characteristics, n = 155

Variable	Median, (range) or number			
	**Exposed**			**Unexposed**
	
	**All** **(n = 116)**	**Formerly** **(n = 70)**	**Currently** **(n = 46)**	**(n = 39)**

Age (years)	55 (37-75)	58 (37-75)	46 (38-64)	60 (41-74)
Height (cm)	179 (166-193)	180 (167-193)	178 (166-190)	178 (170-192)
Weight (kg)	86 (62-161)	86 (64-116)	86 (62-161)	80 (63-135)
Cigarettes smoker	24	14	10	3
Alcohol ≥ 14 units/week	8	4	4	2
Rheumatic disease	0	0	0	1
Thyroid disease	3	2	1	1
Nocturnal symptoms (numbness/tingling)				
*Right hand*	28	17	11	6
*Left hand*	29	19	10	7
Pain (wrist)				
*Right hand*	27	18	9	2
*Left hand*	20	14	6	4
Clumsiness (difficulties in button clothing)				
*Right hand*	29	20	9	4
*Left hand*	26	17	9	3

### Exposure assessment

The cumulative hand-arm vibration dose was calculated as the product of self-reported occupational exposure, as collected by questionnaire and interviews, and the measured or estimated hand-arm vibration exposure in 1987, 1992, 1997, 2002, and 2008. In the calculations, the exposure during the periods between the investigations has been estimated based on values from the latest study. The assessment of vibration exposure was made under normal working conditions with standardized equipment and methods [9] by measuring the intensity of vibration on a random selection of the tools used by the manual workers in accordance with international standards [10,11]. The total number of tools included in the study was 306 and during each investigation period the number of tools that measurement were conducted on varied between 45 and 128, corresponding to between 50% and 90% of the total number of tools used at the workshop. For hand-held tools with two handles, measurements were made on both handles and the highest measured vibration intensity was used in the analysis. The most commonly used tools were grinders and hammers and their mean frequency-weighted acceleration values have decreased over the investigation period from 5.8 to 4.5 m/s^2 ^and 11.0 to 7.6 m/s^2^, respectively [12].

The subjective assessments of daily exposure time were collected by questionnaire and interview. In the questionnaire, the workers were asked to estimate the amount of time (minutes per day) they were exposed to vibration while using the different types of hand-held vibrating tools during their last working day. In the interview workers who had been exposed before 1987 or ended exposure before 1987 were questioned about their use of hand-held vibrating tools (type, exposure time). The total daily exposure time for vibrating tools has decreased from 108 min in 1987 to 52 min per day in 2008 [12]. Leisure-time exposure (hobbies, snowmobiling, motorcycling, etc.) was not included in this measure.

In the part of Sweden where the plant is located job change is infrequent. When students finish vocational school at approximately 18 years old they often find well-paying employment as manual workers and usually stay in the job as long as possible. Our interviews revealed that occupational exposure to hand-arm vibration usually started at age 16 when most workers were in vocational school. Thus, we used the age 16 as onset of exposure time. In vocational school, the two last years consist mainly of work as a trainee. No worker who had any extended time away from hand-arm vibration exposure returned to exposure again. However, some workers left exposed jobs and some of them did so due to vibration-induced vascular symptoms ("vibration white finger").

The cumulative lifetime hand-arm vibration dose was calculated as the product of self-reported occupational exposure in hours and the squared acceleration of the measured or estimated hand-arm vibration exposure (i.e. dose = a^2^·t; unit m^2^s^-4^h). As an example, a worker using a grinder 3 hours per day and a hammer 30 minutes per day for 7 years at exposure values of 5 m/s^2 ^and 10 m/s^2 ^respectively would have had a dose of 7 years × 220 days/year × 3 hours/day × 5^2 ^(m/s^2^)^2 ^+ 7 years × 220 days/year × 0.5 hours/day × 10^2 ^(m/s^2^)^2 ^= 192 500 m^2^s^-4^h. Those exposed were grouped into exposure quartiles with divisions at Q1 (25th centile), Q2 (median), and Q3 (75th centile). Class 1 includes subjects with hand-arm vibration exposure values from 0 to ≤ Q1; class 2 includes subjects with values > Q1 to ≤ Q2; class 3 includes > Q2 to ≤ Q3, and class 4 includes the subjects with the highest exposure values of > Q3. Class 0 contains unexposed subjects (hand-arm vibration exposure equal to zero) and is set as the reference category. Thus 5 classes of cumulative lifetime hand-arm vibration dose were obtained (Table 3).

**Table 3:** Cumulative lifetime hand-arm vibration exposure dose

Class	n	Cumulative vibration dose (m^2^s^-4^h)		
		**Min**	**Median**	**Max**
		
0	39	0	0	0
1	29	2475	56 320	84 865
2	29	85800	128700	192500
3	29	197120	252648	359680
4	29	365420	566764	857813

Moreover, at the time for nerve conduction measurements, we calculated the current daily energy-equivalent exposure value normalized to an eight-hour reference period (i.e. A(8); unit ms^-2^), in accordance with the European directive for vibration [13]. The subjects were grouped into 4 classes regarding current daily exposure. Class 0 contains not ever exposed subjects and class 1 contains subjects with cumulative vibration hand-arm exposure but no current vibration exposure. Among those with current vibration exposure a division into 2 classes were done. Class 2 includes subjects with hand-arm exposure values from 0 to ≤ Q2 and class 3 includes subjects with values > Q2 (Table 4).

**Table 4:** Current daily vibration exposure value

Class	n	Current daily vibration value, A(8), ms^-2^		
		**min**	**median**	**max**
		
0	39	0	0	0
1	70	0	0	0
2	23	0.41	0.84	1.19
3	23	1.27	1.59	4.12

Unless otherwise indicated, when we refer in the text and tables to "exposed subjects", we mean those subjects who currently are or earlier were exposed to hand-arm vibration and consequently the "unexposed subjects" are those who have never been exposed to hand-arm vibration.

### Nerve conduction test

The nerve conduction measurement was performed in March 2008, during wintertime in Sundsvall, with snow and outdoor temperatures usually below 0°C. The average outdoor temperature in March 2008 was -0.4°C.

To ensure an adequate hand temperature and minimize temperature as a source of error [14,15], the determination of conduction velocity was preceded by a bicycle ergometer test, which has been shown to stabilize fingertip skin temperature at around 34°C [16]. Two consecutive runs of 6 min each were conducted on an electrically braked bicycle ergometer (Siemens-Elema). Men under 45 years of age began at load of 100 W, and after 6 min this was increased to 150 W. The equivalent loads for men over 45 were 50 and 100 W, respectively. Skin temperature was measured using a thermistor (Testo^® ^926, Germany) taped to the tip of digit IV. During the nerve conduction test, the subjects were covered with warm blankets. Some participants could not perform the bicycle ergometer test due to cardiovascular diseases or musculoskeletal problems. Seven subjects, therefore, were only covered with warm blankets and eight subjects did perform the bicycling, but only at a low load. There are mathematical formulas for temperature corrections at low temperatures, but those are based on skin temperature at the wrist and are probably not reliable for skin temperature at the fingertip. 

Nerve conduction measurements were made in both arms and hands using a routine electromyography (EMG) apparatus (Keypoint^® ^Portable, Keypoint Software Version 3.0, Medtronic NeuroMuscular, Denmark). The test was performed by an experienced EMG technician, who was blinded to the results of all other tests. The measurements were made on the second floor in the factory and we experienced some technical problems with electromagnetic interference.

The median nerve motor conduction velocity was determined using surface electrodes for stimulation at the elbow and proximal to the wrist and for recording over the abductor pollicis brevis muscle. The ulnar nerve motor conduction velocity was determined using surface electrodes for stimulation 2 cm proximal to the elbow and proximal to the wrist and for recording over the abductor digiti minimi muscle. The distance between the recording and stimulation electrodes at the wrist was 7 cm. The F-wave latency was measured as the shortest latency obtained with 20 stimuli at the wrist. Sensory conduction velocity (SCV) of the median nerve was determined orthodromically from the third finger to the palm and the wrist, respectively, using surface electrodes mounted at fixed sites in a plastic splint held against the skin over the nerve. The distance between recording and stimulation electrodes at the wrist (palm-wrist) was 60 mm and the corresponding distance at the finger and palm (digit III-palm) was 63 mm. Sural nerve SCV was also measured, in order to control for non-symptomatic polyneuropathy, but due to electromagnetic interference these measurements were unreliable and not analyzed in this study.

### Statistics

All descriptive statistics for the study population and nerve conduction outcome are given as medians and ranges or means and standard deviations or as numbers and percentages. Classification of unexposed and hand-arm vibration-exposed individuals were made according to quartiles. Hand-arm vibration exposure is described according to class as minimum, median, and maximum. To compare nerve conduction, temperature, and age between groups, Student's two sample t-test for independent groups was used. Paired t-test was used to compare an individual's nerve conduction velocities between the right and left hands. A multivariate linear regression model was used to assess the association between nerve conduction outcome and exposure variables. Backward elimination and forward selection procedures were used to verify the multivariate linear regression model. The predictor variables in the model were considered to be of biological importance (age, height, weight, skin temperature, alcohol consumption, smoking, classes of vibration exposure, years since last vibration exposure to date of test). Since cumulative vibration exposure and current vibration exposure partly include the same information, two separate models were considered, one for each vibration exposure. For comparing prevalence of median nerve neuropathy, chi-square test and a variant of Fisher's exact test [17] were used.

P-values < 0.05 was considered to be statistically significant. JMP^® ^7 and SAS 9.2 were used to perform the analyses.

## Results

Descriptive characteristics of the study sample are presented in Table 2. Subjects in the unexposed group and formerly exposed group were older than those in the currently exposed group. The groups did not differ regarding height, weight, or skin temperature during measurements.

The subjects who did not attend the nerve conduction measurements were analyzed for age and life-time cumulative hand-arm exposure and did not differ from the studied subjects.

### Nerve conduction

#### Motor conduction velocity

##### Median and ulnar nerves, distal latency

There were no significant differences in median or ulnar nerve distal latencies in either arm between exposed and unexposed subjects (Table 5), nor between classes with cumulative lifetime exposure or current daily exposure (data not shown).

**Table 5:** Nerve conduction measurements.

		Exposed						Unexposed		95% CI Group difference (Exposed [all] - Unexposed)	p-value*
						
		All		Formerly		Currently					
						
		Mean	SD	Mean	SD	Mean	SD	Mean	SD		
Motor examination											

**Median nerve**											
Velocity (m/s)	right	57.8	5.36	58.5	5.29	56.8	5.46	57.6	6.03	-1.96; 2.43	0.83
	left	60.4	6.08	61.0	6.16	59.8	5.68	61.1	7.02	-3.44; 2.01	0.60
Amplitude (wrist) (mV)	right	7.61	3.28	7.48	2.82	7.83	3.92	8.14	3.26	-1.74; 0.69	0.39
	left	8.95	3.42	8.94	3.38	8.8	3.52	7.70	3.40	-0.08; 2.57	0.06
Distal latency (ms)	right	4.42	0.73	4.40	0.67	4.41	0.83	4.28	0.65	-0.11; 0.39	0.28
	left	4.07	0.53	4.09	0.48	4.07	0.63	4.04	0.71	-0.23; 0.30	0.79
F-latency (ms)	right	25.9	2.08	25.7	1.87	26.2	2.37	26.5	2.11	-1.37; 0.23	0.16
	left	25.8	2.11	25.7	2.07	25.8	2.16	25.5	2.04	-0.50; 1.10	0.46
**Ulnar nerve**											
Velocity (m/s)	right	60.4	7.06	60.4	6.8	60.4	7.55	59.7	6.47	-1.83; 3.23	0.58
	left	64.0	6.52	63.8	6.82	64.2	6.19	63.5	6.39	-2.07; 2.97	0.72
Amplitude (wrist) (mV)	right	11.2	2.30	11.2	2.49	11.3	2.01	11.2	2.18	-0.81; 0.86	0.95
	left	10.4	1.99	10.4	2.04	10.3	1.94	10.4	2.20	-0.85; 0.82	0.98
Distal latency (ms)	right	3.36	0.46	3.35	0.43	3.37	0.51	3.38	0.41	-0.19; 0.13	0.73
	left	3.27	0.45	3.29	0.48	3.25	0.42	3.24	0.43	-0.14; 0.20	0.71
F-latency (ms)	right	26.9	2.26	26.9	2.08	26.9	2.54	27.3	1.81	-1.20; 0.25	0.20
	left	26.4	2.21	26.5	2.16	26.2	2.33	26.4	2.26	-0.93; 0.81	0.89

Sensory examination											

**Median nerve**											
Latency, dig III-palm (ms)	right	1.74	0.20	1.72	0.21	1.75	0.20	1.78	0.23	-0.15; 0.06	0.36
	left	1.71	0.19	1.71	0.21	1.71	0.15	1.74	0.27	-0.15; 0.09	0.63
Amplitude (finger) (μV)	right	15.6	8.49	15.9	8.37	15.2	8.87	11.8	9.86	-0.56; 8.18	0.09
	left	17.8	10.5	18.6	11.5	16.9	9.38	15.8	9.07	-2.40; 6.43	0.36
Latency, palm-wrist (ms)	right	1.58	0.26	1.54	0.24	1.61	0.29	1.64	0.43	-0.25; 0.11	0.45
	left	1.50	0.23	1.45	0.21	1.54	0.25	1.48	0.18	-0.07; 0.11	0.67
Amplitude (wrist) (μV)	right	12.6	7.47	12.8	6.65	12.5	8.61	8.05	6.49	1.51; 7.60	0.004
	left	13.1	8.27	13.6	8.24	12.8	8.35	10.6	5.92	-0.58; 5.54	0.11

*p-value for difference between exposed (all) and unexposed

In the multivariate regression analysis, distal motor latency of the median nerve was associated with skin temperature (right/left hand) and age (left hand). Distal motor latency of the ulnar nerve was associated with skin temperature (right/left hand), and height (right/left hand). Neither the cumulative lifetime exposure nor the current daily exposure contributed to explaining the distal latencies in the multiple linear regression models.

Paired t-test for individual measurements between right and left hands in median nerve gave a mean difference of 0.32 ms (SE 0.04, p < 0.001) and the corresponding figure for the ulnar nerve was 0.09 ms (SE 0.03, p = 0.003). Approximately the same figures apply when analyzing data from exposed and unexposed separately. The right hands had the longer distal latency.

The skin temperature during motor conduction measurements of the median nerve was similar between unexposed (right hand 32.4 ± 4.0°C, left hand 32.4 ± 4.0°C) and exposed (right hand 32.2 ± 3.3°, left hand 32.5 ± 3.2°C) subjects and corresponding skin temperature for the ulnar nerve was also similar between unexposed (right hand 32.3 ± 3.8°C, left hand 32.5 ± 3.9°C) and exposed (right hand 32.3 ± 3.0°, left hand 32.6 ± 3.1°C) subjects.

There were no significant differences in skin temperature between classes of cumulative lifetime exposure or current daily exposure.

#### Sensory conduction examination

##### Median nerve, sensory latency, digit III-palm

**Figures 1:**
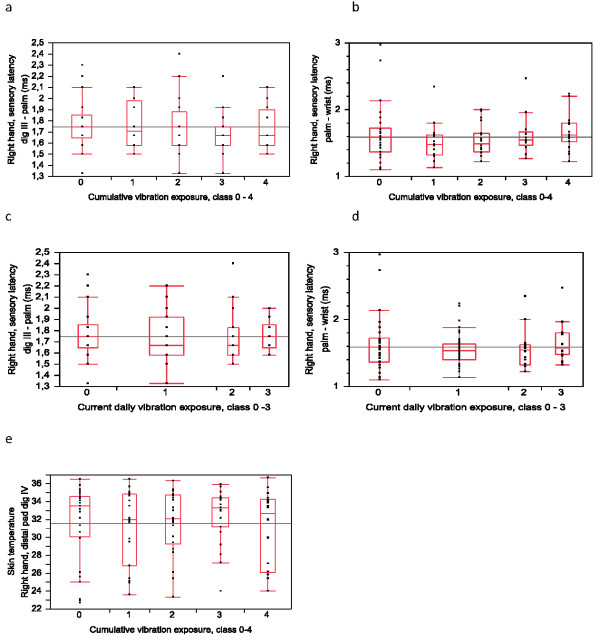
**a-e: Nerve conduction and skin temperature in different classes of vibration exposure**. Median values (-) are presented within the interquartile box. The difference between the quartiles is the interquartile range (Q3-Q1). The whiskers extend to the farthest point that is still within 1.5 interquartile ranges from the quartiles. Grand mean is presented with a line.

There were no significant differences in sensory latencies in either arm between exposed and unexposed subjects (Table 5), nor between classes with cumulative lifetime exposure (Figure 1a) or current daily exposure (Figure 1c).

In the multivariate regression analysis, the sensory latency of the median nerve (digit III-palm) was associated with skin temperature (right/left hand) and age (right/left hand). Neither cumulative lifetime exposure nor current daily exposure contributed to explaining the sensory latency in the multiple linear regression models.

Paired t-test for individual measurements between right and left hands gave a mean difference of 0.03 ms (SE 0.01, p = 0.09). The differential between right and left hands was approximately the same when analyzing data from exposed and unexposed separately, although the p-values were higher (exposed; mean difference 0.02 ms [SE 0.02 p = 0.18] and unexposed; mean difference 0.03 [SE 0.03, p = 0.31]). The right hands had the longer latency.

##### Median nerve, sensory latency, palm-wrist

There were no significant differences in sensory latencies in either arm between exposed and unexposed subjects (Table 5), nor between classes with cumulative lifetime exposure (Figure 1b) or current daily exposure (Figure 1d).

In the multivariate regression analysis, the sensory latency of the median nerve (palm-wrist) was associated with skin temperature (right/left hand). Neither cumulative lifetime exposure nor current daily exposure contributed to explaining the sensory latency in the multiple linear regression models.

Paired t-test for individual measurements between right and left hands gave a mean difference of 0.08 ms (SE 0.03, p = 0.004). When analyzing data from exposed and unexposed separately the paired t-test between right and left hands of the exposed subjects gave a mean difference of 0.06 (SE 0.02, p = 0.006) and the corresponding figure for the unexposed subjects was 0.12 (SE 0.08, p = 0.14). The right hands had the longer distal latency.

Neuropathy of the median nerve at the carpal tunnel segment was considered to be present when the sensory latency from palm to wrist was greater than 1.73 ms at a distance of 60 mm (the cut-off point represents 3SD of the mean value of a normal material collected with similar plastic splint equipment in our laboratory). With this cut-off point there were 15 right hands and 10 left hands with median nerve neuropathy in the exposed group. Corresponding numbers in the unexposed group were 6 and 2. There were 9 subjects with bilateral median nerve neuropathy. Among these 33 hands with median nerve neuropathy, there were 15 hands with one or several of the following symptoms: nocturnal numbness, pain in wrist or fingers, and difficulty in buttoning clothing, reported either in the questionnaire or during medical examination. There were 4 subjects with bilateral symptoms and bilateral median nerve pathology. Presence of median nerve neuropathy with or without symptoms was independent of exposure class (Table 6).

**Table 6:** Neuropathy of the median nerve at the carpal tunnel segment^a^.

	Right hand		Left hand	
	
Cumulative life-time exposure^b^	Nerve conduction n (%)	Nerve conduction + symptoms^c ^n (%)	Nerve conduction n (%)	Nerve conduction + symptoms^c ^n (%)
Class 0	6(23)	3 (12)	2(8)	1(4)
Class 1	2(11)	1(5)	1(6)	1(6)
Class 2	4(19)	0	3(16)	1(5)
Class 3	3(18)	1(6)	3(17)	2(11)
Class 4	6(31)	3(16)	3(17)	2(11)
**Current daily exposure^b^**				
Class 0	6(23)	3(12)	2(8)	1(4)
Class 1	7(16)	2(5)	4(10)	3(7)
Class 2	2(13)	1(7)	2(14)	1(7)
Class 3	6(33)	2(11)	4(23)	2(12)

^a ^Sensory latency from palm to wrist greater than 1.73 ms at a distance of 60 mm

^b ^Results are given as numbers and percentage of measured hands in parentheses.

^c ^Pain and/or nocturnal numbness and/or difficulty in buttoning clothing.

The skin temperature during sensory conduction measurements was similar between unexposed (right hand 31.6 ± 4.3°C, left hand 31.5 ± 4.4°C) and exposed (right hand 31.6 ± 3.7°C, left hand 31.8 ± 3.6°C) subjects. There were no significant differences in skin temperature between classes of cumulative life-time exposure (Figure 1e) or current daily exposure.

In all the above mentioned nerve conduction measurements we have also separately analyzed those subjects with current daily exposure (n = 46) and those with former exposure without current exposure (n = 70) in linear regression models. As the number of the subjects in each group was small, we used fewer predictive variables in the models (age, skin temperature, classes of exposure and "years since last vibration exposure to date of test"). Neither the cumulative exposure nor the current daily exposure or "years since last vibration exposure to date of test" contributed to explaining the nerve conduction measurements.

#### Other nerve conduction measurements

There were no differences in any other measured nerve conduction parameter (conduction velocities, amplitudes, and f-latencies) between unexposed and exposed groups, except for median nerve sensory amplitude at the wrist in the right hand (Table 5). The exposed group had higher amplitude than the unexposed group (12.6 [SD7.5] μV versus 8.1 [SD 6.5] μV), but in the multivariate analysis only age was associated with the amplitude. Neither cumulative lifetime exposure nor current daily exposure contributed to explaining the amplitude in the multiple linear regression models.

### Power statistics

**Figure 2:**
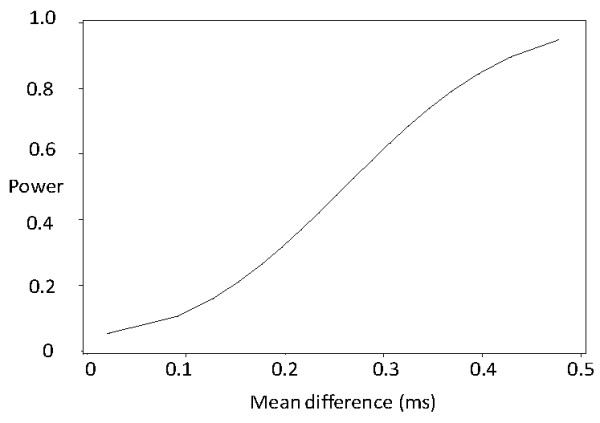
**Power curve, median nerve, distal motor latency, right hand**.

**Figure 3:**
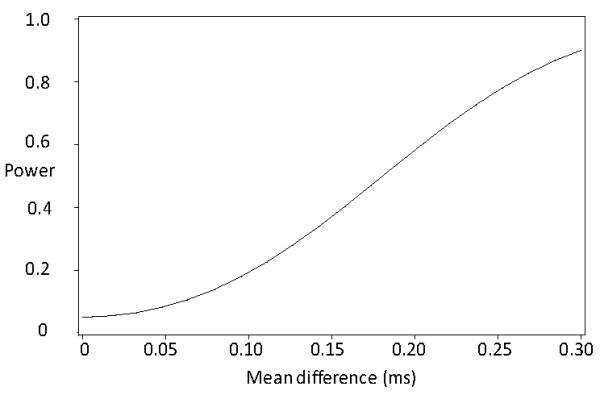
**Power curve, median nerve, sensory latency, palm-wrist, right hand**.

With 80% power we would have been able to detect a difference of 0.38 ms in median nerve distal motor latency in the right hand between unexposed and exposed subjects (Figure 2). Corresponding figures for sensory latency digit III to palm were 0.13 ms and palm to wrist were 0.26 ms (Figure 3). The figures were similar for the left hand, except for the palm to wrist segment where the detectable difference was 0.14 ms.

## Discussion

The strength of this study lies in our careful assessment of subjects' exposure and the consequent reduction of recall bias. In order to minimize the sources of error hand-arm vibration dose was calculated as the product of self-reported occupational exposure, collected by questionnaire and interviews, and the measured or estimated hand-arm vibration exposure in 1987, 1992, 1997, 2002, and 2008. To our knowledge there has been no other study with similar exposure assessment over a period of 21 years. In a recent report, Burström et al concluded that regular surveillance of the exposure and health have significantly reduced the exposure to vibration in this study population [12].

In our electrophysiological study of hand-arm vibration exposed and unexposed subjects, there were no differences between the groups in either the sensory conduction latencies of the median nerve nor in the motor conduction latencies of the median and ulnar nerves. Specifically, exposure to hand-arm vibration was not associated with a decrease of peripheral nerve conduction and we saw no signs of increased slowing in large myelinated fibers. However, one must bear in mind that only the fastest of the large myelinated fibers, and thus a limited portion of the whole nerve fiber population, are examined in nerve conduction studies. Another possibility for the non-positive result in the present study could be that the exposed population is mixed with currently and formerly exposed manual workers and if there exists a recovery factor the mixed population would contribute to diluting the difference between the exposed and the unexposed groups. However, there was no difference in nerve conduction between currently exposed and formerly exposed subjects and the attempt to adjust for a recovery time factor in the regression model by using "years since last vibration exposure to date of test" as a predictor did not contribute to explaining the results of the nerve conduction measurements. Subjects in the unexposed group and formerly exposed group were older than those in the currently exposed group. There was no one older than 64 years in the currently exposed group and 13 (33%) older than 64 years in the unexposed group. We controlled for age in the multiple linear regression models, and it did not alter the fact that vibration exposure was not a predictor of nerve conduction variables in the equation. We also conducted a regression analysis after excluding all subjects with age over 64 years and still the exposure variables did not contribute to explaining the nerve conduction measurements.

The exposed group had higher amplitude than the unexposed in the sensory conduction measurements at the wrist, but in the linear regression model the vibration exposure did not contribute to the model and the difference is probably due to other factors.

When comparing each individual's right and left hand; the right hand had longer distal latency in the motor conduction of the median and ulnar nerves and also slightly longer latency in the sensory conduction of the median nerve over the carpal segment. However, although not significant, the latency difference over the carpal segment was larger in the unexposed subjects. The right hand is generally more exposed to hand-arm vibration in this cohort [18]. The majority is right-handed and the ergonomic load in the workplace and at home is probably higher on this side [19]. Nathan et al.[20] reported slowing in the dominant hand in a prospective study of median nerve sensory conduction in industrial workers, but could not reveal any correlation with occupational hand use.

Seven subjects (9%) and 11 (7%) hands of those who underwent sensory nerve conduction measurements in the exposed group had both pathological sensory nerve conduction at the wrist and symptoms suggestive of carpal tunnel syndrome (CTS); the corresponding numbers in the unexposed group were 3 (12%) and 4 (8%). There was no significant difference between groups. We excluded subjects who had had surgery for carpal tunnel syndrome. If those subjects were included in the calculation, there would still be no difference between exposed and unexposed subjects. The overall prevalence of CTS in the present study is higher than that reported among men in an epidemiological study of the general population in Sweden (2.1%) [21]. A review of occupational populations showed a wide range in the prevalence of CTS (0.6%-61%) [22]. In the present study there was also a high proportion of pathological nerve conduction velocities in the palm-wrist segment in subjects without symptoms. Among those subjects, there were still no differences between exposed and unexposed. This has also been reported in other studies [21,23,24]. Atroshi et al. [21] found abnormal nerve conduction without symptoms to be more common among older subjects. The mean age of the subjects with abnormal nerve conduction in the present cohort was 56 years (range 39-71) and the mean age of the study group was 55 (37-75) years.

Temperature is an important source of error in nerve conduction studies. This was obvious in the study of Cherniack et al.[8], who reported that the significant differences in digital sensory conduction velocities between vibration-exposed and non-exposed workers, which had been observed after segmental cutaneous warming, were eliminated after systemic warming with a bicycle ergometer test. Moreover, the strong association between increased skin temperature and faster sensory conduction velocities, which had been observed after segmental cutaneous warming, was largely eliminated for both digital and palmar anatomic segments after systemic warming. We had hoped to increase the temperature before the nerve conduction measurements by using the bicycle ergometer test, which had previously proved to be an effective method to increase the skin temperature of the fingers [16], but our effort to raise the skin temperature in fingertips failed in some cases. However, the skin temperature was only measured at the fingertip of digit IV and it is possible that the skin temperature was higher at the wrist. Hence, we had a number of subjects with skin temperatures at the fingertip below 32°C. On the other hand, there were no differences in mean skin temperature between the exposed and unexposed or between classes with cumulative life-time exposure or current daily exposure. We chose to control for temperature in the multiple linear regression model, and it did not alter the fact that vibration exposure was not a predictor of nerve conduction variables in the equation. We also conducted an analysis after excluding all subjects with finger temperature under 32°C and there were still no differences in skin temperature or nerve conduction between classes of vibration exposure.

At baseline in 1987 the present cohort was investigated with nerve conduction measurements in a cross-sectional study; Nilsson et al.[25] reported impaired nerve conduction in the exposed group. The risk was not proportional to the vibration exposure. They concluded that the contributions from vibration and ergonomic factors to the impaired nerve conduction were inseparable. We do not know why the difference between unexposed and exposed is not detectable 21 years later. Possible reasons could be recovery due to retirement, job transfer, or due to fewer or less vibrating tools and/or decreased daily exposure time; another reason, that different methods were used in the two studies for measuring nerve conduction velocity e.g. in the present study we used a systemic warming method to eliminate the temperature as a source of error. A third, possibility is that those who had impaired nerve conduction in 1987 are among those we have not been able to follow-up, and finally, a fourth reason could be lack of power to detect a small difference in nerve conduction.

Our results, with no differences in nerve conduction velocity between hand-arm vibration exposed and unexposed subjects differ from the results of several other epidemiological studies. Most of the studies that demonstrate an association between vibration exposure and nerve conduction impairment come from case-control studies where the vibration-exposed workers have been selected either from a population of patients, subjects with suspected hand-arm vibration syndrome disorders [2,4,26], or from job categories entailing a well-recognized exposure to vibration [27-29].

In our present study, the majority of the sample does not have severe neurological symptoms and most subjects have not been referred to a clinic. Based on the present results we propose that nerve conduction velocity may not be a sufficiently sensitive method for detecting small hand-arm vibration-related pathological changes in peripheral nerves.

### Limitations of the study

Although there appears to be little difference between the 197 invited subjects and the final study group, the reduction in our sample size weakens the statistical power of our analyses, i.e. the ability to reject the null hypothesis of no differences. Thus we would caution that a relationship between hand-arm vibration exposure and peripheral neuropathy may exist but has not been detected in this study.

In occupational studies one must also bear in mind the healthy worker effect, i.e. exposed workers who develop symptoms of peripheral neuropathy leave their jobs and are not selected for future studies of working populations. This type of selection bias would probably lead to a negative bias in the estimation of the effect of hand-arm vibration exposure on peripheral neuropathy. However our study sample was from a working population where the workforce turnover, to our knowledge, was low and the study population had no extreme disability rate.

Epidemiological studies have indicated an association of carpal tunnel syndrome and ergonomic factors such as high requirements for hand force, prolonged work with extended wrist, high repetitiveness, and their combination. In this study we did not control for these factors.

## Conclusions

Nerve conduction measurements of peripheral hand nerves revealed no exposure-response association between hand-arm vibration exposure and distal neuropathy of the large myelinated fibers in a cohort of male office and manual workers.

## Abbreviations

HAVS: Hand-arm vibration syndrome; T.N: Tohr Nilsson; EMG:electromyography; SCV: sensory conduction velocity; CTS: carpal tunnel syndrome.

## Competing interests

The authors declare that they have no competing interests.

## Authors' contributions

HS wrote the manuscript, contributed to the design of outcome measurements, performed the statistical analysis and the interpretation of data. AJ discussed and contributed to the manuscript, participated and contributed substantially to the analysis and interpretation of data. BGW discussed and contributed to the manuscript, contributed to the design of outcome measurements and participated and contributed substantially to the interpretation of data. LB discussed and contributed to the manuscript, designed the study, was principal investigator and data collector of the exposure measurements, participated and contributed substantially to the analysis and interpretation of data. RL discussed and contributed to the manuscript, designed the study, participated and contributed substantially to the analysis and interpretation of data. TN discussed and contributed to the manuscript, designed the study, was the examining physician at the baseline and the follow ups, participated and contributed substantially to the analysis and interpretation of data. MH initiated and designed the study, discussed and contributed to the manuscript. All authors have read and approved the final manuscript.
